# MicroRNAs: Biological Regulators in Pathogen–Host Interactions

**DOI:** 10.3390/cells9010113

**Published:** 2020-01-02

**Authors:** Stephanie Maia Acuña, Lucile Maria Floeter-Winter, Sandra Marcia Muxel

**Affiliations:** Department of Physiology, Universidade de São Paulo, 05508-090 São Paulo, Brazil; stephanie.acuna@usp.br (S.M.A.); lucile@usp.br (L.M.F.-W.)

**Keywords:** microRNAs, gene expression, post-transcriptional, pathogen, parasite, host, immune response

## Abstract

An inflammatory response is essential for combating invading pathogens. Several effector components, as well as immune cell populations, are involved in mounting an immune response, thereby destroying pathogenic organisms such as bacteria, fungi, viruses, and parasites. In the past decade, microRNAs (miRNAs), a group of noncoding small RNAs, have emerged as functionally significant regulatory molecules with the significant capability of fine-tuning biological processes. The important role of miRNAs in inflammation and immune responses is highlighted by studies in which the regulation of miRNAs in the host was shown to be related to infectious diseases and associated with the eradication or susceptibility of the infection. Here, we review the biological aspects of microRNAs, focusing on their roles as regulators of gene expression during pathogen–host interactions and their implications in the immune response against *Leishmania*, *Trypanosoma*, *Toxoplasma*, and *Plasmodium* infectious diseases.

## 1. Introduction

The discovery of noncoding RNAs (ncRNAs) has revolutionized the field of molecular biology. These ncRNAs do not code for proteins but globally impact genome maintenance and gene expression [1,2,3,4]. These RNAs can be categorized according to their length, localization, and function, such as ncRNAs that regulate gene expression, as long noncoding RNAs (lncRNAs), microRNAs (miRNAs), small interfering RNAs (siRNAs), and PIWI-interacting RNAs (piRNAs); RNA maturation, as small nucleolar RNAs (snoRNAs) and small nuclear RNAs (snRNAs); and protein synthesis, as ribosomal RNA (rRNAs) and transfer RNAs (tRNAs) [5,6].

miRNAs (typically 21 nucleotides) are a class of ncRNAs which interact with the 3′ untranslated region (3′ UTR) of messenger RNAs (mRNAs), leading to mRNA degradation or translational repression [7,8,9,10]. The involvement of miRNAs in a variety of biological processes, such as chronic pathologies and infectious diseases, illustrates their complexity as they have been described as being able to simultaneously interact with diverse molecules, such as RNA binding proteins (RBPs), making the miRNA recognition site more accessible to the RNA-induced silencing complex (RISC), which also enables the processing of pri-miRNAs [11,12] or lncRNAs [1,13], controlling different points of the gene expression flux.

Host–pathogen interactions result in signaling and physiological modifications in host cells that induce the miRNA-mediated post-transcriptional regulation of genes involved in the inflammatory response during the induction of the immune response [14,15]. These miRNAs are involved in the modulation of both innate and adaptive immune responses [16]. In recent years, the alteration of miRNA expression has been studied extensively in cancer or infectious diseases caused by bacteria, viruses, and parasites [17,18,19,20].

The proposition of miRNAs as potentially non-invasive biomarkers for clinical diagnosis of diseases, using patient plasma, has prompted descriptions of tumor-derived miRNAs in that medium [21,22]. Also, some studies have shown that miRNAs are differentially expressed in patients with hepatotoxicity [23], artery disease [24], and infectious diseases [19,20,25].

In this review, we provide an overview of the miRNA regulation of different biological processes in pathogen–host interactions, focusing on the interactions caused by protozoan parasite infections, such as *Leishmania* spp., *Trypanosoma* spp., *Toxoplasma* spp., and *Plasmodium* spp., and expanding the possibilities for regulation of the gene expression of inflammatory immune responses.

The emergent miRNomics field, including new databases and computational tools, could lead to new approaches for studying host–pathogen interactions by offering insights from vertebrate and invertebrate host miRNA, as well as pathogen miRNAs.

## 2. miRNAs—Biogenesis and Gene Expression Regulation

**Figure 1:**
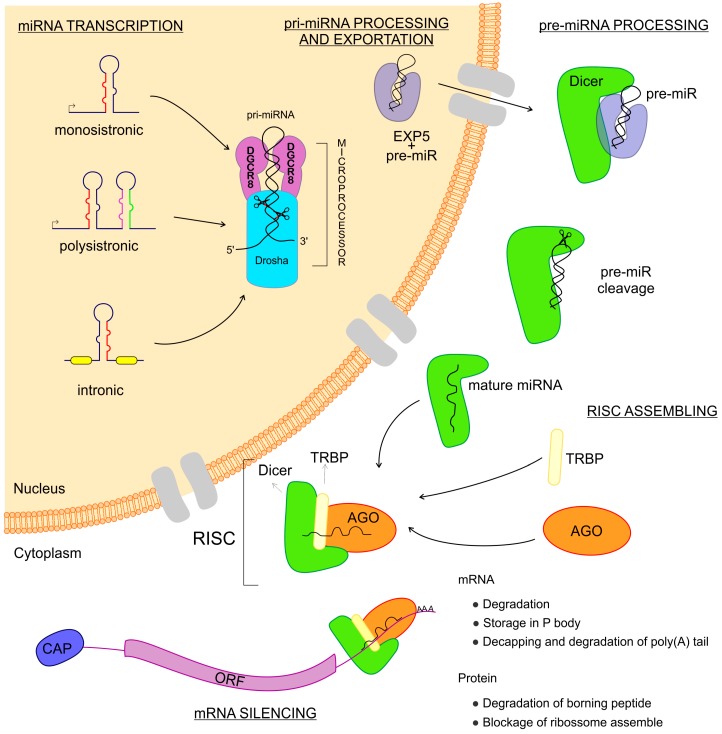
Biogenesis of microRNAs (miRNAs). The miRNAs are small non-coding RNAs transcribed from DNA sequences which can be monocistronic or polycistronic, comprised within exons, introns, or a unique host gene. Right after the transcription, this new RNA sequence is called primary-miRNA (pri-miRNA), which is folded in a hairpin conformation and coupled with the microprocessor: a combination of DGCR8 and Drosha RNases which cut the pri-miRNA, making it a pre-miRNA. Afterward, the pre-miRNA is coupled to Exportin 5 and then exported to the cytoplasm where this complex is found by Dicer, which cuts the pre-miRNA, releasing two mature miRNA arms. The mature miRNA complexed to the Dicer can now couple with the Argonaute and TRBP proteins, thus assembling the RNA Induced Silencing Complex–RISC. When the RISC is done, it can find the messenger RNAs that are the targets to the miRNA complexed. Once found, the message is now silenced and the gene expression regulated.

miRNAs are transcribed from intergenic regions as either monocistronic or polycistronic (miRNA-clusters) transcripts [26,27,28]. Those sequences can have their own promoter region or can depend on the transcription of host genes if they are intragenic [26,27,28]. They can be encoded in exonic or intronic regions and transcribed in the same direction as that of the pre-messenger RNA, leading to the use of the promoter region of mRNAs for their transcription [29] (Figure 1). miRNAs are transcribed by RNA polymerase II and fold into long double-strand primary miRNA transcripts (pri-miRNA) [7]. In the nucleus, the class 2 RNase III DROSHA and DGCR8 (a double-strand RNA-binding protein, also known as Pasha) complex recognizes features in the hairpin structures of pri-miRNA and processes the molecule to form the precursor miRNA transcript (pre-miRNA) [30,31]. The pre-miRNA is coupled with the Exportin 5 protein and exported to the cytoplasm [32] where an RNase III family protein Dicer complexed with TRBP (transactivation response element RNA-binding protein) recognizes and processes the pre-miRNA into the miRNA-duplex, a mature miRNA [33,34]. The functional strand of the mature miRNA is loaded into the RISC, coupled with the argonaute (AGO) protein family as important components of ribonucleoprotein (RNP) complexes (miRNPs) which guide interactions with the target mRNA, leading to the regulation of gene expression [33,35,36,37].

miRNAs regulate gene expression at a post-transcriptional level in a sequence-specific manner, exerting a massive biological impact [7]. Research has been dedicated toward attempting to understand the mechanisms of post-transcriptional regulation mediated by miRNAs, with studies performed in vitro, in vivo, and in cell-free extracts, as well as using bioinformatics prediction tools to demonstrate the putative regulation, by miRNAs, of nearly 30% of all protein-coding genes in mammalian cells [38]. miRNAs can regulate translation by (a) repressing the initiation of protein translation of 7-methylguanosine (m^7^GpppN)-capped mRNAs, inhibiting the binding of the eukaryotic translation initiation factor (eIF) subunits eIF4E, eIF4F, and eIF4G, which promote the scaffolding of mRNA for association of the ribosome initiation complex [39,40,41]; (b) preventing the association of ribosome 60 S subunit with 40 S and mRNA via interference of the binding between eIF6 and 60 S, which can be mediated by the AGO2–Dicer–TRBP complex [42]; and (c) blocking elongation via the miRNA–mRNA association with active polysomes, impacting the post-initiation step of translation [43]. miRNAs can also regulate mRNA destabilization by recruiting decay machinery components, leading to mRNA poly(A) tail deadenylation by exonuclease activity in 3′→5′ degradation, or decapping followed by 5′→3′ exonuclease activity [44,45]. In addition, miRNAs are secreted into vesicles, as exosomes, or into extracellular fluids and circulation, highlighting the potential roles of extracellular miRNAs in mediating intercellular communications and as biomarkers for a variety of diseases, including infectious diseases [46].

## 3. miRNA Expression in Infectious Diseases

### 3.1. Leishmania–Host Interactions

Leishmaniases are vector-borne diseases that are caused by more than 20 different species of the protozoa parasite *Leishmania* in humans [47]. They consist of a large spectrum of clinical manifestations, from cutaneous manifestations, where symptoms remain localized to the skin or mucosal surfaces, to visceral lesions after the migration of the parasite to internal organs such as the liver, spleen, and bone marrow [48,49]. This disease is considered a neglected tropical disease, endemic in 98 countries around the world, with the annual incidence for cutaneous leishmaniasis estimated to be 0.7–1.2 million and 0.2–0.4 million for visceral leishmaniasis [47,50]. *Leishmania* has a dimorphic life cycle, alternating between extracellular replicative promastigote forms that live in the digestive tract of the sand fly (*Phlebotomus* and *Lutzomyia*) and amastigote forms that live in the interior of the phagolysosomal compartment in mammalian host phagocytic cells, such as macrophages, neutrophils, and dendritic cells (DCs) [50,51]. The initial steps of the inflammatory response occur during the phagocytosis of *Leishmania* and its establishment inside the phagolysosome and it is able to modulate the immune response by reducing the efficiency of inflammation and the development of an adaptive immune response [52,53,54,55]. Recent studies demonstrated that infections caused by *L. amazonensis*, *L. infantum*, *L. major*, and *L. donovani* can induce alteration in the miRNA profile from macrophages and dendritic cells of humans, murines, and dogs, thereby implicating the recognition and activation mechanisms of the immune response against parasites [56,57,58,59,60,61,62,63].

The modulation of the immune response is an intricate network that is essential in defining the fate of infection by *Leishmania*, regulating the polarization of T CD4^+^ lymphocytes for both type 1 (Th1—pro-inflammatory) or type 2 (Th2—anti-inflammatory) infection [55,64,65,66], in which macrophage polarization is also implicated. Cytokines and chemokines, such as interferon gamma (IFN-γ), tumor necrosis factor alpha (TNF-α), and granulocyte macrophage colony-stimulating factor (GM-CSF) and/or the Toll-like receptor (TLR) ligand, are characteristic of Th1-cells and responsible for differentiating macrophages into the M1 phenotype, which increases nitric oxide synthase 2 (NOS2) expression and NO production with leishmanicidal activity [67,68,69,70]. On the other hand, Th-2 cytokines and chemokines, such as interleukin 4 (IL-4), IL-13, tumor growth factor beta (TGF-β), IL-10, and macrophage colony-stimulating factor (M-CSF), differentiate macrophages into the M2 phenotype, which expresses higher levels of arginase 1 (ARG1), favoring parasite survival [67,68,69,70,71,72]. Some of the miRNAs modulated during infection have been implicated in macrophage polarization, as shown for the M1 phenotype (miRNA-146 and miRNA-210) and for M2 phenotype (miRNA-130a, miRNA-130b, miRNA-155, miRNA-21, miRNA-19a, miRNA-23a, miRNA-125a, miRNA-125b, miRNA-26a, miRNA-26b, and miRNA-720) [73]. In this way, the transcription profile of murine macrophages indicates a mix of M1/M2 phenotypes in murine macrophages infected with *L. amazonensis,* whereas in BALB/c macrophages, differential downregulation of Il1b is observed once C57BL/6 macrophages upregulate Il1b, a molecule that mediates nitric oxide (NO) production and confers resistance against *Leishmania* infection [74,75].

**Figure 2:**
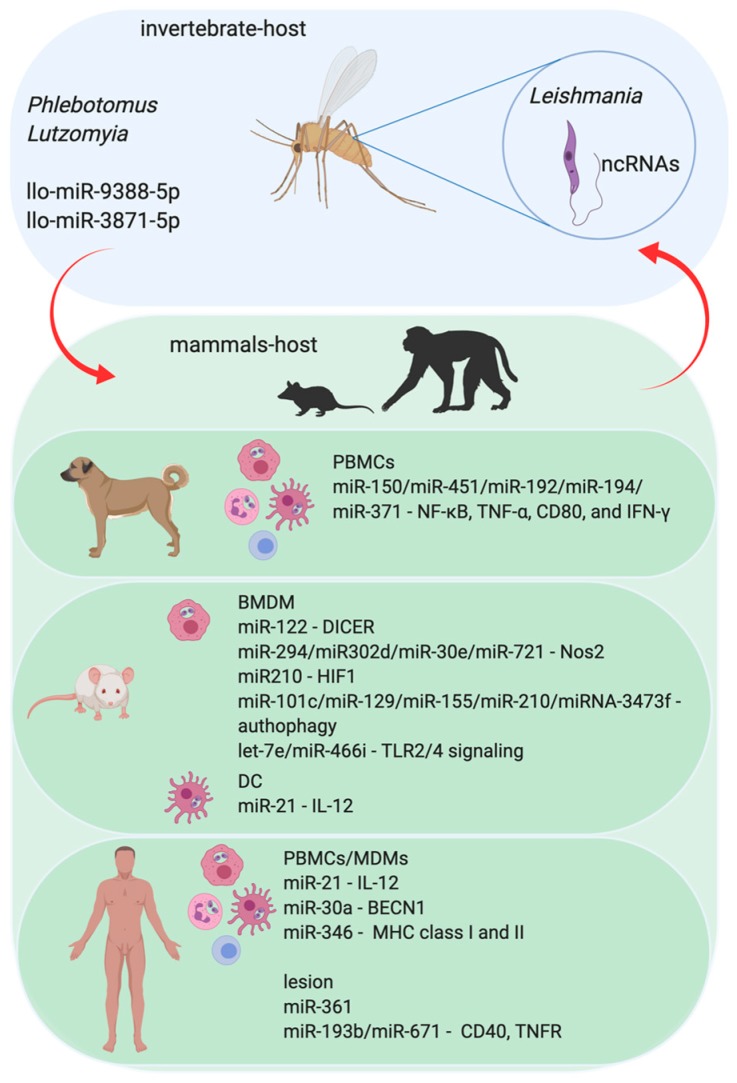
The role of miRNA in the *Leishmania* parasite and parasite–host interaction. The protozoan infection caused by *Leishmania* parasite leads to Leishmaniasis in mammalian hosts. The infection begins with the transmission during the blood meal of infected female sandflies (*Phlebotomus* and/or *Lutzomyia)* and the consequent injection of infective promastigotes into the host’s epidermis. Flagellated and mobile extracellular promastigote form of *Leishmania* proliferate in the gut of sandflies. Upon penetration, promastigotes are identified by immune phagocytic cells, such as macrophages, neutrophils, and dendritic cells (DC). Once the parasites are phagocytized by these immune cells, they are differentiated into amastigote forms and begin to multiply within those cells. Amastigotes have a reduced flagellum and ovaleted morphology and survive inside the phagolysosomal compartment. Parasites in promastigote form express noncoding RNAs (ncRNAs) that can regulate the invertebrate host–sandfly–metabolic pathways. However, sandflies also express miRNAs, which can affect the parasite biology, influencing its success in the colonization of sandflies’ digestive tract and differentiation to the infective forms and the vertebrate host. Not only do the invertebrate hosts express miRNAs, but the vertebrate one also does. The experimental models using human Monocytes Derived-Macrophages (MDM) or murine Bone Marrow-Derived Macrophages (BMDM), and also in the Peripheral Blood Cells (PBMCs) from human and dogs show that infection of mammalian hosts can interfere with the profile of host miRNAs. Also, the plasma samples or the cutaneous lesion site from leishmaniasis patients show modifications in the miRNA profile.

Interestingly, some virulence factors of parasites interfere with the miRNA machinery, regulating host mRNA expression. As observed for *L. donovani*, the parasite glycoprotein gp63 targets the host Dicer1, cleaving Dicer to downregulate pre-miR-122 and its processing to miR-122, resulting in post-transcriptional regulation of host mRNA/miRNA interactions, leading to increased parasite burden in mouse liver [59], as shown in Figure 2. The activity of *L. amazonensis* arginase, an enzyme that uses l-arginine to produce ornithine and urea, can impact the regulation of macrophage miRNA expression during infection [61]. The use of l-arginine by both host and parasite arginases could alter the amino acid availability for host activation of the microbicidal response once l-arginine is used by NOS2 to produce NO [61]. Indeed, l-arginine deprivation leads to the downregulation of the CD3ζ chain in T cells, thereby suppressing the T cell response [76,77]. Indeed, miR-122 repression of the cationic amino acid transporter (CAT1) can regulate intracellular l-arginine availability [78]. Besides, miR-155 represses the expression of arginase 2 in DC, preventing l-arginine depletion and allowing the activation of T cells [79].

In addition, parasites can affect the activation of microbicidal mechanisms, such as NO production, and also cell recruitment to the location of the lesion. Our group showed that *L. amazonensis* induces upregulation of miR-294-3p and miR-721 in BALB/c-BMDM, which binds to *No*s2 3′UTR, reducing the levels of NOS2 and NO production and increasing infectivity [61]. Also, miRNAs that are deregulated during infection, such as miR-30e and miR-302d, interfere with *Nos*2 mRNA expression and NO production; miR-294 and miR-302d regulate *Tnf* mRNA levels and miR-294 alters *Ccl*2/*Mcp*-1 mRNA, implicating the expression of these miRNAs in controlling infectivity [80]. Indeed, for a set of miRNAs, let-7a, miR-25, miR-26a, miR-132, miR-140, miR-146a, and miR-155, their upregulation in *L. major*-infected human macrophages was negatively correlated with the expression of their corresponding chemokine targets, CCL2, CCL5, CXCL10, CXCL11, and CXCL12, thereby corroborating the data that indicate the downregulation of chemokines CCR2, CCL5, and CXCL10 during infection [81]. Likewise, the activation of hypoxia inducible factor-1 (HIF-1α) partially controls miR-210 during the *L. major* infection of human macrophages, linking HIF-1α overexpression to susceptibility to both *L. donovani* and *L. amazonensis* [82]. This mechanism occurs via the downregulation of the NF-κB mediating transcription of pro-inflammatory cytokine genes, such as TNF-α and IL-12, thereby altering the immune response to parasite survival inside the macrophages [83]. Interestingly, miR-21 is upregulated in peripheral blood mononuclear cells (PBMCs) infected with *L. donovani*, and miR-blocking increases the expression of IL-12 mRNA in murine-DCs infected with *L. donovani*, leading to a proliferation of CD4^+^ T cells [84]. Further, miRNA-361-3p, a regulator of TNF, was observed to be negatively correlated with localized cutaneous leishmaniasis (LCL) skin lesions caused by *L. braziliensis*, which is resistant to pentavalent antimonial and healing of parasites [85], while the expression of miR-193b and miR-671 was observed to be correlated with that of their respective target genes, CD40 and TNFR, in lesions of *L. braziliensis*-infected patients.

Furthermore, canine visceral leishmaniasis in symptomatic dogs who were naturally infected with *L. infantum* showed a differential modulation in the expression of miR-150, miR-451, miR-192, miR-194, and miR-371 in PBMCs, which can target genes to regulate the immune response and pathogenesis, such as NF-κB, TNF-α, CD80, and IFN-γ, which are important molecules related to resistance against the disease [62]. The *L*. *infantum* infection of human U937 and THP-1-derived macrophages upregulates miR-346, which decreases the mRNA level of genes initially associated with decreased MHC class I and II antigen presentation, TAP1, RFX1, and BCAP31.

Parasites can incite anti-inflammatory responses and modify antigen presentation by deregulating the host’s autophagy machinery via mimicking apoptosis signals, such as those known as apoptotic-like *Leishmania*, and reducing the proliferation of CD4 T cells [86,87]. The miRNAs miR-101c, miR-129-5p, miR-155, and miR-210-5p, which are differentially expressed in *L. major*-infected BMDM, were related to the activation of the autophagic machinery and interfere with parasite clearance [56]. The *L. donovani* infection of THP-1 and human-MDM upregulates miR-30a levels and its inhibition increases the autophagic mechanism via negative regulation of Beclin 1 (BECN1), thereby decreasing infectivity [88]. Likewise, the *L. donovani*-infected murine macrophage expression of miRNA-3473f inhibits autophagy [63].

Moreover, miR-511 is upregulated in DCs infected with *L. donovani* and can interfere in Toll-like receptor 4 (TLR4) signaling, which is an important molecule in the recognition and activation of the immune response to *Leishmania* infections [89]. Also, *L. donovani* strain, which is resistant to sodium stibogluconate, a pentavalent antimonial that is used for visceral leishmaniasis treatment in the Indian subcontinent, upregulates the expression of miR-466i in murine macrophages that target MyD88, increasing the levels of IL-10 production and the severity of disease [57]. Our group showed that the upregulation of let-7e, let-7f, and let-7g occurs in a MyD88, TLR2, or TLR4 dependent way during *L. amazonensis* infection and let-7e inhibition increases the expression of *NOS2* mRNA, the NOS2 protein amount, and NO production, thus impacting infectivity [90]. Also, let-7e inhibition during *L. amazonensis* infection upregulates the levels of the validated targets, *Tnfpaip*3, *Map*2*k*4, *Tbk*1, and *Tnf*, as well as in the predicted targets, *Traf*6, *Ppara*, *Mapk*8*ip*3/*Jip*3, *Map*3*k*1, and *Ube*2n, globally impacting TLR signaling gene expression [90]. It is interesting that the infection of macrophages with *Mycobacterium* and *Neisseria* upregulate the levels of let-7e [91,92]. Indeed, let-7e targets p65 NF-κB activation and phosphoinositide-3 kinase/serine–threonine protein kinase (Pi3k/Akt) and TLR4, reducing the expression of pro-inflammatory cytokines, such as TNF, IFN-α, IL-6, and the chemokines MCP-1, MIP-1, and IP-10.

Some studies corroborate the integration of the transcription factors and miRNA regulation of host immune responses. *L. donovani* increases the levels of the c-Myc transcription factor in human MDMs, leading to an upregulation of DROSHA and a downregulation of miRNAs let-7a, miR-151, miR-34a, miR-98, miR-148b, and miR-378a, while c-Myc knockdown reverts the expression regulation pattern of these molecules and attenuates parasite survival [93]. c-Myc overexpression sustains M2 polarization, a characteristic of human tumor-associated macrophages (TAMs), and regulates the expression of vascular endothelial growth factor (VEGF), matrix metallopeptidase 9 (MMP9), HIF-1α, and transforming growth factor beta (TGF-β).

Geraci and coauthors elegantly showed that let-7a, let-7b, and miR-103 are upregulated in DCs and macrophages that are infected with *L. donovani*, but downregulated in *L. major* infections [60]; miRNAs that target genes involved in inflammation and their expression can also modulate interferon regulation, demonstrating the importance of *Leishmania* species in determining the outcome of an infection and the miRNA profile. Overall, *Leishmania* species can induce a wide-ranging variance in the miRNA profile, which is correlated with the target transcript and host cell types, supporting species-specificity in miRNA regulation and the host recognition of pathogen guides miRNA–mRNA interactions during *Leishmania* infection.

Regarding the invertebrate host, phlebotomine sandflies are well recognized as vectors for species of *Leishmania* and a wide range of pathogens, such as bacteria and viruses, with the regulation of miRNAs favoring pathogen survival and transmission [94,95,96]. Yang and Wu discriminated miRNAs from other noncoding RNAs in *Lutzomya longipalpis,* a vector of leishmaniasis in the Americas and also suggest the role of miRNA targeting genes involved in *Leishmania* infection, such as llo-miR-9388-5p and llo-miR-3871-5p [97] in *Phlebotomus perniciosus,* a major vector of *Leishmania infantum* in Europe [98].

Computational algorithm prediction and RNA-seq analysis support the evidence of ncRNAs in *Leishmania,* as shown for *L. amazonensis*, *L. braziliensis*, *L. donovani*, *L. infantum*, and *L. major*, indicating that ncRNA may have a role in gene expression modulation and could help us understand genomic organization, as well as transcriptional expression and regulation in promastigote and amastigote forms. However, only *L. braziliensis* possesses a functional RNAi pathway and AGO1 [99,100,101,102].

### 3.2. Trypanosoma–Host Interactions

Chagas disease is another neglected tropical disease caused by the parasite *Trypanosoma cruzi*. It is a significant public health problem, affecting 6 to 7 million people worldwide (WHO) [103]. Although the impact of Chagas disease has decreased over the years, it is still a public health problem in Latin America. Additionally, the disease has been described in non-endemic areas, such as Canada, the United States, Europe, Australia, and Japan, mainly due to its transmission by blood transfusion, organ transplantation, vertical transmission, or accidental ingestion [103,104,105,106]. In South America, *T. cruzi* is mainly transmitted by the feces of infected Triatominae bugs during their blood-meal [103,104,105,106]. The disease is characterized by parasite invasion in the bloodstream, infecting many cell types in different tissues, including muscle cardiac cells. The disease presents two clinical phases, acute and chronic, varying from asymptomatic with non-specific symptoms to cardiomyopathy, dysfunction of the digestive tract, or alterations in the nervous system [107,108]. In both phases, there is an activation of the immune system. In the acute phase, the immune system acts to control the infection and parasite replication. By contrast, during the chronic phase, several cascades are regulated and related to the autoimmune and/or to the inflammatory response sustained by parasite persistence [109]. The progression and severity of the disease can vary according to the individual, geographic region, and/or parasite tissue tropism, presenting specific genotype and phenotype characteristics that can affect host–pathogen interactions [110]. Benznidazole and nifurtimox, the treatments that are currently available, began to be used over 40 years ago. Moreover, both drugs have several limitations, such as long treatment times and being primarily effective for acute forms of disease [111]. Thus, new effective therapies and prognosis markers are required.

Gene expression profiling of myocardial tissue and the heart have shown differential gene expression to be modulated among chronic Chagas patients, as well as acute and chronic *T. cruzi*-infected mice. The differentially expressed genes are related to immune response, energy and metabolism, and cell stress response [112]. The inflammatory response comprises the activation of the TLRs cascade signaling, the induction of IFN-γ production in T cells [112,113], and antibody production by B cells to reduce parasitaemia, which is counterbalanced by IL-10. Despite an innate and adaptative response, low-grade chronic infection was established to infiltrate Th1 cells, thereby producing IFN-γ in myocardial tissue and leading to heart damage [112,114,115].

The involvement of miRNAs in Chagas disease has also been described to act in the gene expression modulation of physiological and pathophysiological factors. The interplay between heart tissue cells, including cardiomyocytes, fibroblasts, endothelial, and infiltrating inflammatory cells, and the possibility of miRNA transfer across these cells represents a complex connection between miRNAs and mRNAs, impacting the resistance to infection and pathogenesis.

**Figure 3:**
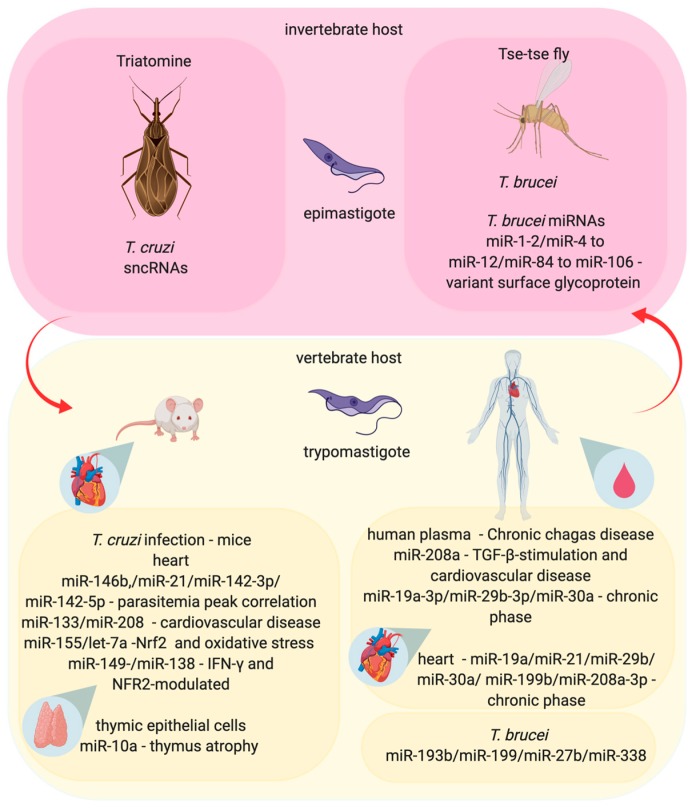
The influences of miRNA in the interaction between the Trypanosoma, vector, and mammalian host in Chagas disease and Sleep sickness. The *Trypanosoma cruzi* causes a Chagas disease and is transmitted during the Triatomine bug blood-meal, when the bug defecates and transfers trypomastigotes form to the vertebrate host. The itchy skin, due to the bug’s bite, allows the trypomastigote form to enter in the dermis, where the parasite infects any cell, differentiates into amastigote form, and replicates inside the infected cells, causing the clinical symptoms of the disease. *T. cruzi* express small noncoding RNAs (sncRNAs) that can change the regulation of gene expression inside the invertebrate host (Triatomine). Also, *T. cruzi* infection can modify miRNAs expression in the human and murine heart-infected cells, in the murine thymic epithelial infected cells or circulating miRNAs in human plasma samples, impacting cardiomyopathy, parasitemia and the immune response. On the other hand, sleep sickness is caused by *T. brucei.* The parasite is transmitted during the blood-meal of infected tsetse flies, which inject trypomastigote form into the vertebrate host. Different from the *T. cruzi* infection, *T. brucei* trypomastigotes circulate in the blood, lymph, and spinal fluid and can cross the blood brain barrier to infect the central nervous system. This parasite expresses miRNAs correlated with the modulation of the expression of virulence factors, such as variant surface glycoproteins. The miRNA profile is altered in plasma samples of patients infected with *T. brucei*.

The modulation of miRNAs from the hearts of *T. cruzi*-infected mice is positively correlated with a parasitemia peak at 30 days post-infection (dpi), as observed for miR-146b, miR-21, miR-142-3p, and miR-142-5p, while a negative correlation is observed for miR-145-5p and miR-149-5p and also suggests the regulation of genes involved in the pathophysiological conditions of experimental infection, such as calcium and potassium channels and electrocardiography (ECG) parameters [116], as shown in Figure 3. The dysregulation of miR-133 and miR-208 have been correlated with heart genes that are related to cardiovascular disease in chronic Chagas patients and acute *T. cruzi*-infected mice [112,114,115]. Ferreira et al. showed the upregulation of miRNAs in whole heart tissue, such as miR-155-5p in the acute (15 dpi) and chronic phase (30 and 45 dpi) and let-7a-5p in the chronic phase of infection with *T. cruzi* in the murine experimental model. This pattern of miRNA modulation was correlated with Nrf2 transcriptional response and oxidative stress [117]. In addition, miR-149-5p, miR-138-5p, and miR-16-5p appeared to be differentially expressed during *T. cruzi* infection through modulation of IFN-γ and NFR2-modulated genes in infiltrating cells and, consequently, the control of parasitism and tissue damage [117].

Higher levels of miR-208a in the plasma samples from human chronic Chagas disease were described to be correlated with TGF-β-stimulation and the regulation of genes involved in cardiac hypertrophy and fibrosis [118,119,120]. Nonaka and coworkers also found increased levels of miR-19a-3p, miR-29b-3p, and miR-30a-5p in plasma and miR-19a-3p, miR-21-5p, miR-29b-3p, miR-30a-5p, miR-199b-5p, and miR-208a-3p in heart samples from chronic Chagas patients, which was correlated with miR-21 upregulation in TGF-β-stimulated fibroblasts [121]. These miRNAs can regulate distinct genes and mechanisms, such as miR-193b targeting of TGF-β2 [122], apoptosis by miR-338 targeting of apoptosis-associated tyrosine kinase (AATK), and can also be induced by pro-inflammatory cytokines, such as those observed for TNF-α and IL-6 stimulation, that upregulate miR-199a in human adipocytes [123].

The infection of B6 mice by *T. cruzi* leads to increased levels of miR-10a in thymic epithelial cells (TEC) involved in intrathymic T cell differentiation, which can be caused by TGF-β signaling and impact thymus atrophy during infection [124,125].

*T. cruzi* genome and transcriptome analysis showed the presence of ncRNA, such as small nuclear RNA (snRNA), small nucleolar RNA (snoRNA), and transfer RNA (tRNA) [126,127,128] (Figure 3). Indeed, sncRNAs can be delivered into HeLa cells by extracellular vesicles from parasites, increasing the susceptibility to infection [129].

Regarding *T. brucei gambiense* and *T. b. rhodesiense*, these cause a fatal human disease known as African sleeping sickness chronic illness, which is prevalent in West Africa and East Africa, respectively [130]. *T. brucei* is transmitted by the bite of infected tsetse flies (genus Glossina), alternating the bloodstream-form in the mammalian host (man, domestic animals, and ruminants) and insect-form of life [131]. Parasites injected in hosts cause a local inflammatory reaction, a tender reddish swelling, and the multiplication of parasites in the plasma and interstitial fluid begin an acute febrile illness. *T. b. gambiense* classically causes an enlargement of cervical lymph nodes and can progress to neurological diseases, such as meningoencephalitis, lethargy, and coma. Besides, *T. b. rhodesiense* mostly causes acute systemic diseases, such as haemolymphatic alteration, swollen lymph nodes, fever, and rapid weight loss. *T. brucei* resides in the bloodstream and evades the host immune response by antigenic variation [131,132,133]. Interestingly, the miR-193b and miR-338 were upregulated in blood samples from patients and miR-199a-3p and miR-27b were seropositive for *T. b. gambiense* [134]. The *T. brucei* transcriptome profile identified ncRNAs, and a description of these molecules could improve our knowledge of parasite biology [135,136]. Interestingly, *T. brucei* present a total of 881 predicted miRNAs, including the genomic cluster of miRNAs that codify identical precursors, such as miR-1-2 and miR-4 to miR-12, as well as miR-84 to miR-106, which can impact virulence factors, such as variant surface glycoprotein (VSG), in different stages of the transformation and proliferation of parasites [137]. However, *T. brucei* possesses a functional RNAi as well as AGO1 machinery, but the functional RNAi machinery was not found in *T. cruzi*, *L. major*, and *L. donovani* [99].

### 3.3. Toxoplasma–Host Interaction

*Toxoplasma gondii* is a ubiquitous parasite that can infect a large range of hosts, including mice, humans, pigs, birds, sheep, and cats, the last of which is the definitive host where sexual replication takes place. Its life cycle is complex depending on the host. When it replicates asexually, the parasite presents two forms: tachyzoites and bradyzoites—a fast replicant and a slow replicant, respectively. In addition, bradyzoites can encyst and remain dormant for a long period of time until the host becomes immunocompromised [138]. Infection in the main range of hosts starts with the ingestion of raw infected meat or the ingestion of oocysts that are shed in cat feces. The parasite then starts its odyssey of conquering as many cells as possible and replicating. *T. gondii* is a highly successful parasite due, in part, to its silent colonization of its host [139,140]. Almost all types of immune cells are recruited to control infection with *T. gondii:* macrophages, neutrophils, dendritic cells, lymphocytes, and NK cells [139,141,142,143,144]. Although, almost all infections are silent and this parasite is able to change the host’s miRNA profile, modifying the way that it responds to the infection. Moreover, the parasite also presents its own miRNA processing machinery and miRNAs.

**Figure 4:**
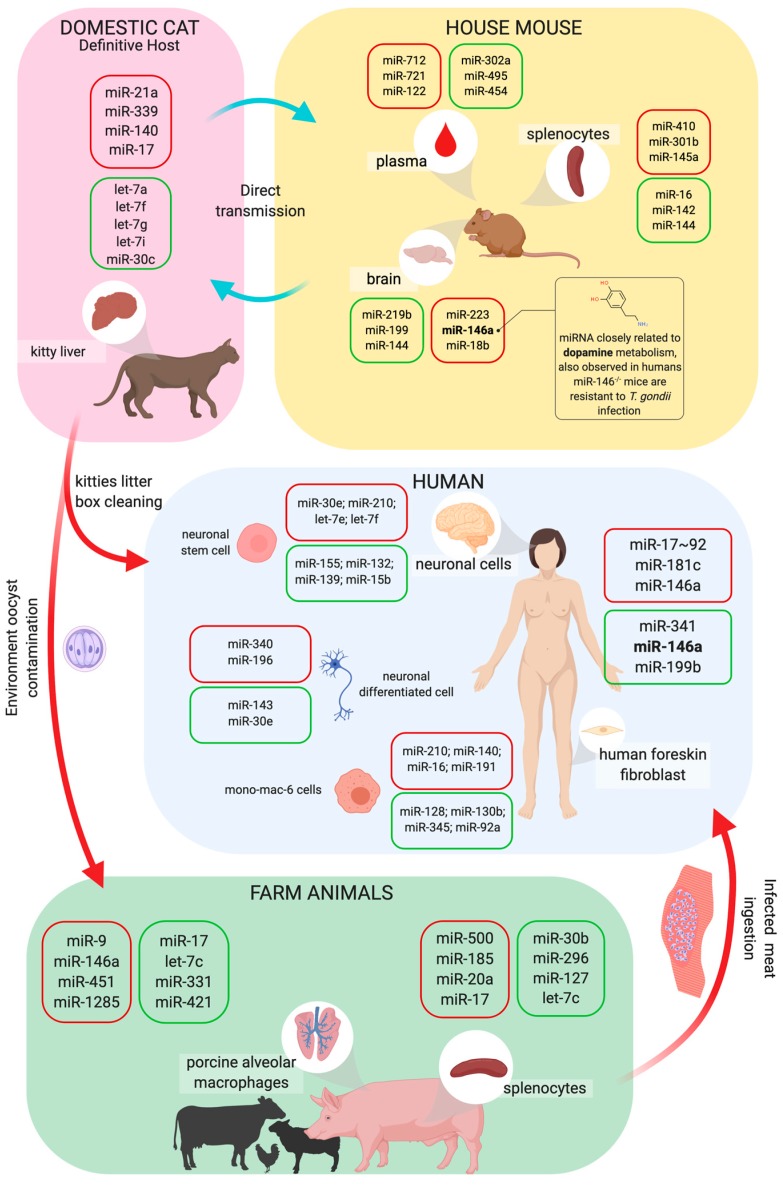
Life cycle of *Toxoplasma* and host miRNA interaction. *Toxoplasma gondii* has a complex life cycle, in which the parasite can infect a large range of animals, such as domestic cats, farm animals, mice, and even humans. Cats are the *T. gondii* definitive host. Oocysts are released from infected cats’ feces, sporulate in the environment, and become infective. Samples of kittens’ liver present modifications in miRNA expression. Humans can also become infected by eating undercooked meat of infected animals or while nursing infected cats. In the human host, this infection can lead to toxoplasmosis, a disease that affects various organs’ tissues such as skeletal muscle, myocardium, and the brain. As a result, neuronal cells, monocyte/macrophage, and fibroblasts from infected patients present modifications in miRNA expression. Also, porcine alveolar macrophages and splenocytes samples have altered miRNA profiles in infected pigs. To study toxoplasmosis in vivo, mice models are commonly used because mice can naturally be infected by the parasites and affect multiple organs while changing their miRNA profile. Mice are largely used to study the disease outcomes and treatments, they can also be naturally infected and have multiple organs affected with the infection, and it is already known that the spleen, plasma, and brain have their miRNA profile changed during the infection. The box color represents the increase (red) or decrease (green) of miRNAs expression.

The first published study on this subject, released in 2010, correlated the miRNA profile of primary HFFs cells (human foreskin fibroblasts) with *T. gondii* infection. This study demonstrated the upregulation of two clusters: miR-17-92 (miR-17, miR-18, miR-19a, miR-20a, miR-19-b1, and miR-92-1) and miR-106b-25 (miR-106b, miR-93, and miR-25) in infected cells [145], as shown in Figure 4. Interestingly, part of the miRNAs in both clusters share the same seed sequence, suggesting that these miRNAs are related with the regulation of the same kinds of processes in HFF cells. [145]. Later, in 2014, another group showed that the expression of the miR-17~92 cluster in human macrophages is dependent on STAT3 (signal transducer and activator of transcription) and these miRNAs can regulate apoptosis due to the inhibition of BIM, which is a pro-apoptotic molecule [146]. This mechanism for inhibiting apoptosis is a known pathway that enables the *T. gondii* to evade an immune response [147]. There are many studies on the role of the miR-17-92 cluster in relation to several cellular processes, including cancer, the immunopathogenesis of autoimmunity, inflammation, or even chronic diseases. This cluster is encoded in humans via MIR17HG (miR-17 host gene) [148,149]. This open reading frame has its transcription regulated by c-Myc [150], HIF1 [149], and many other transcription factors related to inflammation. In addition, the functions of this miRNA cluster are related with proliferation as well as B and T cell differentiation [149,151]. miR-17~92 are found to regulate immune cell proliferation. B and T cells are examples of this phenomenon [149,151]. miR-17~92 helps T cells to differentiate, proliferate, and polarize into T helper cells [151], however this cluster is also related to the differentiation of regulatory T cells, as described by Xiao et al. in 2008, who demonstrated that a mutation in the mice *miR17hg* host gene leads to lymphoproliferative disease and autoimmunity because of a decrease in the differentiation of this kind of cell [152]. For B cell biology, the conditional deletion of the Dicer in early B cell progenitors blocked the differentiation of pro-B cells to pre-B cells and upregulated miR-17~92 targets, especially the pro-apoptotic Bim molecule [153], suggesting, again, that this cluster is related to apoptosis regulation.

Although miR-17-92 and miR-106-25 are not the only miRNAs that are modified when infection with a parasite is established, many other studies have demonstrated that hosts can have their miRNA expression patterns modified. *Toxoplasma* infection in pigs has been described with global miRNA changes in cells infected with the parasite—one showing the changes in splenocytes infected with the YZ-1 Chinese strain (type IX) and the other showing changes in alveolar macrophages. Comparing the type I and type II strains, both assessed the miRNA profile by sequencing small RNAs. Between these three *Toxoplasma* strains, miR-17 presented different patterns of regulation, for example, when porcine alveolar macrophages (PAMs) were infected with the RH strain, their expression was downregulated. On the other hand, infection with the Me49 strain upregulated the expression of this miRNA. In addition, when porcine splenocytes were infected with YZ-1, miR-17 was upregulated after 25 dpi [154]. However, PAMs also presented changes in the miRNAs that regulate TNFα, indicating a predictive link between some novel miRNAs. Thus, it is practical to imagine that this inflammatory pathway might be modified due to infection, since the more silent the infection, the more successful it is, and TNFα signaling leads to an inflammatory response that can destroy all parasites. It is also interesting to observe that in chronic infection of pig spleen (50 dpi), there is a massive downregulation of miRNAs.

Mice are the main study models for many infectious diseases such as toxoplasmosis. Thus, understanding how these animals respond to infection remains of interest. For example, Canella et al. used paired tests between infected HFF and mice (in vivo) to compare different *Toxoplasma* strains, with different virulence results [155]. They observed that miR-146a was upregulated in HFF infection with Me49 and downregulated in infection with the RH strain. They also demonstrated that the expression of ROP16 was closely related to the upregulation of that miRNA. They also demonstrated that the knockout of miR-146a made the animal resistant to a virulent *Toxoplasma* strain and the deletion of ROP16 increased the expression of miR-146a in a strain-specific fashion, thereby demonstrating that there is a balance between parasite protein expression and the host miRNA profile. *T. gondii* RH and Me49 strain infections of BALB/c mice upregulated miRNAs miR-712-3p, miR-511-5p, and miR-217-5p in the host plasma in a specific way as these miRNAs are not found in samples from mice infected with *Plasmodium berghei*, *P. yoelii*, *P. chabaudi*, *Cryptosporidium parvum,* mouse hepatitis virus (MHV), or *Staphylococcus aureus* [156]. Also, Jia and coworkers proposed using circulating miRNA as biomarkers for the detection of *T. gondii* infection, even in the early phase of infection [156].

Infected mice brains were also studied to compare the global miRNA changes during infection with *T. gondii* PRU strain oocysts during acute and chronic infection [157]. This kind of comparison was also made using the spleen of mice infected with the *T. gondii* RH strain, comparing the acute and chronic phases of infection [158]. In addition, the human neuroepithelial cell line was infected with RH-2F, PRU, or CTG to compare how those strains could modify the miRNA profile of neuronal cells, as well as the normal functions of neurons. It was observed that the upregulation of miR-132 during infection with all studied strains was responsible for regulating the expression of genes involved in dopamine metabolism [159]. In 2017, a North America-based cohort study revealed that infection with *T. gondii* affects the human brain in many pathways related to epilepsy, neurodegeneration, and cancer. This study presented the complex interaction between the proteomic and miRNomic profiles in the brain of congenitally infected patients and the following strains: type I—RH and GT1; type II—Me49 and PRU; and type III—VEG. The authors also evaluated the polymorphisms involved in the susceptibility/resistance to infection in the families of these patients. They observed that NF-κB and TGFβ were the central nodes of the complex net of interactions among all those molecules; the authors also observed allelic variants in *NFκB* and *TGFβ* among the studied families [160].

Changes in the miRNA profiles of cats were also an object of study since cats are the definitive host of *Toxoplasma*. The global miRNA profile of cat liver infected with the PRU *Toxoplasma* strain (type II) showed 82 modulated miRNAs, with 48 downregulated. Moreover, miR-17 appeared to be upregulated, demonstrating that this miRNA/cluster expression is closely related to infection with type II strains of *T. gondii* [161].

### 3.4. Plasmodium–Host Interactions

Malaria is an infectious disease that is caused by *Plasmodium* spp. parasites and remains the most devastating illness in tropical countries, which is responsible for the death of over a million people every year, mainly in sub-Saharan Africa, Asia, and Latin America [162]. Children under 5 years of age are the most affected [162]. The parasite life cycle involves the infection of humans through the bites of infected female *Anopheles* mosquitoes who inject sporozoite forms that migrate to the liver, thereby infecting hepatocytes. Rupture of the infected hepatocytes initiates the asexual intraerythrocytic cycle [162]. Hepatosplenomegaly is a hallmark of malaria and the *Plasmodium* asexual stage causes this pathology and subverts protective immunity, whereas splenocytes and liver Kupffer cells are able to remove/eliminate senescent and infected red blood cells (iRBCs) [163,164]. Splenic macrophages are the principal elements in the clearance of malaria-causing parasites from the blood-stage and have been the focus of many studies concerning protective immunity; nevertheless, miRNA profiles in these populations of cells have not been studied. However, the miRNA expression in plasma, liver, and cerebral samples are well studied [165,166,167].

Several studies have shown changes in the miRNA profile in infection by P. falciparum and *P. vivax,* which cause malaria in humans, as well as in experimental malaria models of self-healing *P. chabaudi* infections of mice and *P. berghei* ANKA, which causes cerebral malaria in mice and alters the development of acquired protective immunity against *Plasmodium* [165,168,169]. Chloroquine therapy, a drug which is recommended for malaria treatment, alters the miRNA profile and reduces the levels of NOD-like receptor (NLR) family pyrin domain-containing 1 and 3 (NLRP1 and NLRP3) genes related to inflammasome signaling [170].

**Figure 5:**
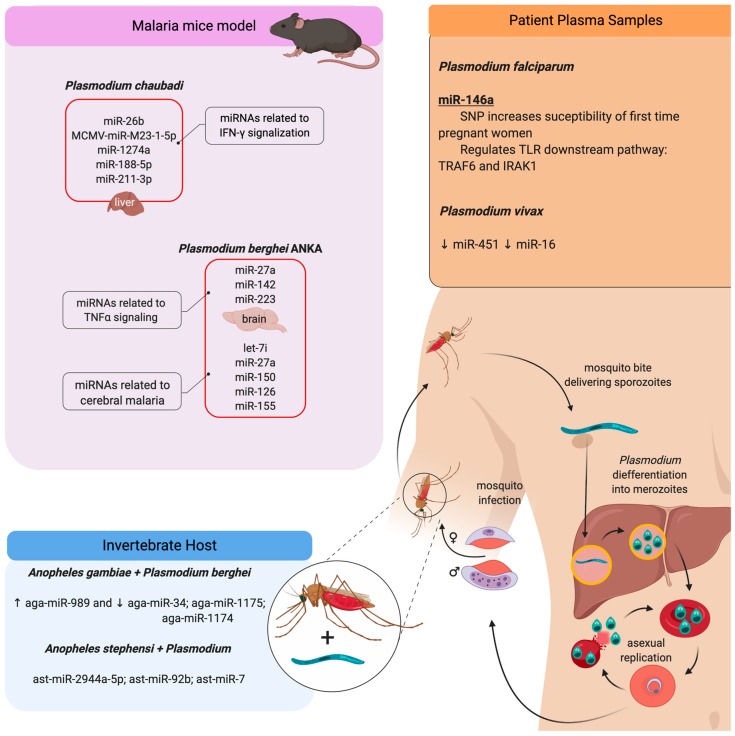
*Plasmodium* induces miRNA profile modifications during pathogen–host interaction. Human malaria infection begins with the bite of infected female mosquito *Anopheles* (invertebrate host), delivering sporozoite forms that migrate to the liver and differentiate into merozoites. These forms leave the liver and start the blood-stage asexual replication. In this stage, *Plasmodium* can differentiate into gametocytes, which can be ingested by the mosquito and develop the sexual phase of the life cycle. Afterward, the mosquito can infect another individual. The species *P. falciparum* and *P. vivax* are anthropophilic, infecting only humans; both of them can induce modifications in the miRNA profile in human plasma samples. Besides, mice infections with *P. chaubadi* and *P. beghei* have been used as malaria experimental models. In the murine infection, the parasite changes the circulating miRNA profile and it is seen in the same way in the liver of infected mice with *P. chaubadi* as it is seen in the brain of mice infected with *P. berghei* ANKA. Although, the invertebrate host presents modifications in the miRNA profile when infected with *Plasmodium*. The red boxes represent the upregulation of miRNAs related to these models. The arrows represent the upregulation (↑) or downregulation (↓) of miRNA.

The levels of miR-451 and miR-16 are downregulated in plasma samples from patients with *P. vivax* infection [169]. However, the miR-146a rs2910164 polymorphism, which could affect the expression level of mature miR-146a, is associated with increased susceptibility to *P. falciparum* infection in first-time pregnant women [171]. miR-146a is involved in the regulation of molecules of TLR signaling, such as TNF receptor-associated factor 6 (TRAF6) and IL-1 receptor-associated kinase 1 genes (IRAK1) [172] (Figure 5).

The *P. chabaudi* infection of mice is an experimental model that shares some characteristics with *P. falciparum* [173,174,175,176] and helps us understand the immune response and pathogenesis in malaria infection, as well as the generation of protective immunity that is related to T and B cell activation and pro-inflammatory cytokine production, such as TNF-α and IFN-γ [176]. *P. chabaudi* infection upregulates miR-26b, MCMV-miR-M23-1-5p, and miR-1274a in the liver, which is correlated with the induction of the immune response, as characterized by the expression of pro-inflammatory cytokines IL-1β, TNF-α and IFN-γ, and NF-κB [177]. *P. berghei* infection deregulated miR-21, miR-122, and miR-155, and ectopic upregulation of miR-155 in liver-resident macrophages (Kupffer cells) activates IFNγ- and TNF-associated pathways [165]. *P. chabaudi* mice infection promotes an increase of miR-188-5p, miR1187, miR-1196-5p, miR-211-3p, miR-32-3p, miR-3082-5p, miR-3960, miR-466i-5p, miR-468-3p, miR-574-5p, miR-669n, miR-709, mir-5126, and miR-6538 in the liver during the acute phase of infection [166], which is characterized by IFN-signaling activation, leading to the activation of the inflammasome and programmed cell death [178,179]. Also, vaccination blocks the decrease in the expression levels of let-7, miR-122-5p, miR-142-3p, miR-148-3p, miR-26a-5p, miR-27a-5p, miR-29b-3p, miR-2861, miR30a/c-5p, miR-3968, and miR-5097, which contribute to liver regeneration [166].

Cerebral malaria in *P. berghei* ANKA-infected mice increases the levels of miR-27a, miR-142, and miR-223 when compared to *P. yoelii*-infected mice; these miRNAs are implicated in TNF signaling and monocyte sequestration in cerebral microvessels in cerebral malaria [167]. The upregulation of let-7i, miR-27a, miR-150, miR-126, miR-210, and miR-155 are correlated with immunomodulator signals, apoptosis, and leukocyte adhesion, and are characteristic of the pathogenesis of experimental cerebral malaria.

Some ncRNAs are described in the malaria parasite *P. falciparum*, which differs in expression during different stages of the parasite life cycle, which is associated with regulation of the expression of virulence-related genes, such as the *var* gene family [180,181].

For invertebrate hosts, interesting studies have shown the involvement of miRNAs as important regulators of *Anophele*s resistance against *Plasmodium* infection. The *P. berghei*-infected midgut of *A. gambiae* showed an increased level of aga-miR-989 and a reduction in aga-miR-34, aga-miR-1175, and aga-miR-1174 [182,183]. Also, the Drosha, Dicer I, and Argo1 knockdown strategy favored *P. berghei* survival [183]. Indeed, sugar-feeding of A. *stephensi* upregulated miRNAs, such as ast-miR-263a, ast-miR-283, and ast-miR-210 [184]. Moreoever, *A. stephensi* feeding of parasite-infected mammals altered the expression of the following miRNAs: ast-miR-2944a-5p, ast-miR-92b, ast-miR-989, ast-miR-275, ast-miR-281-3p, ast-miR-281-5p, ast-miR-306, ast-miR-263a-5p, ast-miR-7, ast-miR-309, and ast-miR-305-3p [184].

## 4. Novel Perspectives: MicroRNAs as Diagnostic Markers and Therapeutic Targets

miRNAs have been emerging as biomarkers and treatment assets of many diseases [185,186]. Why not think about them as a profitable approach to better diagnose and properly treat parasitic diseases? Here, we revised how miRNAs are related to parasitic diseases and included not only the vertebrate hosts, but also the invertebrate ones. Moreover, parasitic diseases affect animals that provide economical and emotional benefits to people. It is inarguable that the loss of support pets or livestock animals causes socio-economic and psychological consequences for people. Nevertheless, a deep gap persists in the development of therapeutic strategies for neglected diseases.

Although, there is an increasing number of studies that are being done in miRNA, however translational research of miRNA still remains a challenge. Earlier this year, Hanna et al. reviewed the role of miRNAs in clinical studies. They observed that findings in miRNA research are limited to academia, often do not make it through phase 3 or 4 of clinical trials, and there is a gap between basic science and clinical application [185,186].

Regarding the neglected diseases, researchers have dedicated decades to the development of new drugs and identifying new biomarkers of disease progression. Despite this, the use of miRNAs as a biomarker and its potential for treatment are poorly explored when compared with hotspot diseases like cancer, cardiac and circulatory diseases, and neurological disorders. Currently, there are only two clinical trials regarding parasitosis and both are cohort studies. The first one was developed in Chicago city, USA, correlating the miRNA profile with the protein expression in toxoplasmosis patients [160]. The other study is focused on figuring out what miRNAs are modified in plasma samples from patients who suffer from acute myocarditis caused by the Chagas disease from Salvador city in Brazil. This study is available in the clinical trials database to consult (<https://clinicaltrials.gov/ct2/show/NCT01842880?term=miRNA&cond=Trypanosomiasis&draw=2&rank=1>).

Thus, the myriad of processes regulated by miRNAs makes them a very profitable approach to be studied, not only as biomarkers, but also as drugs in the treatment of pathologies [186]. Diversity of cell culture platforms, animal models, and the evaluation of circulating fluids (saliva, plasma, and serum) are now available to assess the treatment mechanisms, toxicity, and potential therapeutic efficacy of miRNA candidates in vitro [185,186]. Also, miRNAs can be used in the association of conventional treatments to target a large number of genes and pathways that deliver the host susceptibility to disease and ameliorate the prognosis. Further, the impact of specific miRNAs or a combination of some miRNAs on the regulation of immune responses in the hosts remains a significant challenge.

## 5. Conclusions

The distinct regulation of miRNA–mRNA interaction during host–pathogen interactions highlights the importance of species-specificity, cell type, and a host’s genetic background in determining the details of post-transcriptional regulation of gene expression mediated by miRNAs. Nevertheless, the regulation of the same miRNAs, such as miR-146a and/or miR-155, modulated in host cells that are infected with *T. gondii* infection, cerebral malaria, and *L.* *major* infection [56,81,165] leads to the identification of the signature of the host cell response to parasites and can help in the identification of common characteristics that are implicated in the subversion of the immune response.

## References

[1] Fernandes J.C.R., Acuna S.M., Aoki J.I., Floeter-Winter L.M., Muxel S.M. (2019). Long non-coding RNAs in the regulation of gene expression: Physiology and disease. Non-Coding RNA.

[2] Long Y., Wang X., Youmans D.T., Cech T.R. (2017). How do lncRNAs regulate transcription?. Sci. Adv..

[3] Derrien T., Johnson R., Bussotti G., Tanzer A., Djebali S., Tilgner H., Guernec G., Martin D., Merkel A., Knowles D.G. (2012). The gencode v7 catalog of human long noncoding RNAs: Analysis of their gene structure, evolution, and expression. Genome Res..

[4] Djebali S., Davis C.A., Merkel A., Dobin A., Lassmann T., Mortazavi A., Tanzer A., Lagarde J., Lin W., Schlesinger F. (2012). Landscape of transcription in human cells. Nature.

[5] Ulitsky I., Bartel D.P. (2013). LincRNAs: Genomics, evolution, and mechanisms. Cell.

[6] Rinn J.L., Chang H.Y. (2012). Genome regulation by long noncoding RNAs. Annu. Rev. Biochem..

[7] Bartel D.P. (2004). MicroRNAs: Genomics, biogenesis, mechanism, and function. Cell.

[8] Bagga S., Bracht J., Hunter S., Massirer K., Holtz J., Eachus R., Pasquinelli A.E. (2005). Regulation by let-7 and lin-4 miRNAs results in target mRNA degradation. Cell.

[9] Lim L.P., Lau N.C., Garrett-Engele P., Grimson A., Schelter J.M., Castle J., Bartel D.P., Linsley P.S., Johnson J.M. (2005). Microarray analysis shows that some microRNAs downregulate large numbers of target mRNAs. Nature.

[10] Ben-Hamo R., Efroni S. (2015). MicroRNA regulation of molecular pathways as a generic mechanism and as a core disease phenotype. Oncotarget.

[11] Nussbacher J.K., Yeo G.W. (2018). Systematic discovery of RNA binding proteins that regulate microRNA levels. Mol. Cell.

[12] Hao J., Duan F.F., Wang Y. (2017). MicroRNAs and RNA binding protein regulators of microRNAs in the control of pluripotency and reprogramming. Curr. Opin. Genet. Dev..

[13] Yu Y., Nangia-Makker P., Farhana L., Majumdar A.P.N. (2017). A novel mechanism of lncRNA and miRNA interaction: Ccat2 regulates mir-145 expression by suppressing its maturation process in colon cancer cells. Mol. Cancer.

[14] Baltimore D., Boldin M.P., O’Connell R.M., Rao D.S., Taganov K.D. (2008). MicroRNAs: New regulators of immune cell development and function. Nat. Immunol..

[15] O’Neill L.A., Sheedy F.J., McCoy C.E. (2011). MicroRNAs: The fine-tuners of toll-like receptor signalling. Nat. Rev. Immunol.

[16] Labbaye C., Testa U. (2012). The emerging role of mir-146a in the control of hematopoiesis, immune function and cancer. J. Hematol. Oncol..

[17] Manzano-Roman R., Siles-Lucas M. (2012). MicroRNAs in parasitic diseases: Potential for diagnosis and targeting. Mol. Biochem. Parasitol..

[18] Petrini E., Caviglia G.P., Abate M.L., Fagoonee S., Smedile A., Pellicano R. (2015). MicroRNAs in hbv-related hepatocellular carcinoma: Functions and potential clinical applications. Panminerva Med..

[19] Reynoso R., Laufer N., Hackl M., Skalicky S., Monteforte R., Turk G., Carobene M., Quarleri J., Cahn P., Werner R. (2014). MicroRNAs differentially present in the plasma of hiv elite controllers reduce hiv infection in vitro. Sci. Rep..

[20] Fu Y., Yi Z., Wu X., Li J., Xu F. (2011). Circulating microRNAs in patients with active pulmonary tuberculosis. J. Clin. Microbiol..

[21] Mitchell P.S., Parkin R.K., Kroh E.M., Fritz B.R., Wyman S.K., Pogosova-Agadjanyan E.L., Peterson A., Noteboom J., O’Briant K.C., Allen A. (2008). Circulating microRNAs as stable blood-based markers for cancer detection. Proc. Natl. Acad. Sci. USA.

[22] Lawrie C.H., Gal S., Dunlop H.M., Pushkaran B., Liggins A.P., Pulford K., Banham A.H., Pezzella F., Boultwood J., Wainscoat J.S. (2008). Detection of elevated levels of tumour-associated microRNAs in serum of patients with diffuse large b-cell lymphoma. Br. J. Haematol..

[23] Ozer J., Ratner M., Shaw M., Bailey W., Schomaker S. (2008). The current state of serum biomarkers of hepatotoxicity. Toxicology.

[24] Fichtlscherer S., De Rosa S., Fox H., Schwietz T., Fischer A., Liebetrau C., Weber M., Hamm C.W., Roxe T., Muller-Ardogan M. (2010). Circulating microRNAs in patients with coronary artery disease. Circ. Res..

[25] Abd-El-Fattah A.A., Sadik N.A., Shaker O.G., Aboulftouh M.L. (2013). Differential microRNAs expression in serum of patients with lung cancer, pulmonary tuberculosis, and pneumonia. Cell Biochem. Biophys..

[26] Treiber T., Treiber N., Meister G. (2019). Regulation of microRNA biogenesis and its crosstalk with other cellular pathways. Nat. Rev. Mol. Cell Biol..

[27] Lai F., Gardini A., Zhang A., Shiekhattar R. (2015). Integrator mediates the biogenesis of enhancer RNAs. Nature.

[28] Lagos-Quintana M., Rauhut R., Yalcin A., Meyer J., Lendeckel W., Tuschl T. (2002). Identification of tissue-specific microRNAs from mouse. Curr. Biol..

[29] Lai E.C., Tomancak P., Williams R.W., Rubin G.M. (2003). Computational identification of drosophila microRNA genes. Genome Biol..

[30] Lee Y., Ahn C., Han J., Choi H., Kim J., Yim J., Lee J., Provost P., Radmark O., Kim S. (2003). The nuclear RNAse iii drosha initiates microRNA processing. Nature.

[31] Han J., Pedersen J.S., Kwon S.C., Belair C.D., Kim Y.K., Yeom K.H., Yang W.Y., Haussler D., Blelloch R., Kim V.N. (2009). Posttranscriptional crossregulation between drosha and dgcr8. Cell.

[32] Yamazawa R., Jiko C., Choi S., Park I.Y., Nakagawa A., Yamashita E., Lee S.J. (2018). Structural basis for selective binding of export cargoes by exportin-5. Structure.

[33] Bernstein E., Caudy A.A., Hammond S.M., Hannon G.J. (2001). Role for a bidentate ribonuclease in the initiation step of RNA interference. Nature.

[34] Hutvagner G., McLachlan J., Pasquinelli A.E., Balint E., Tuschl T., Zamore P.D. (2001). A cellular function for the RNA-interference enzyme dicer in the maturation of the let-7 small temporal RNA. Science.

[35] Schwarz D.S., Hutvagner G., Du T., Xu Z., Aronin N., Zamore P.D. (2003). Asymmetry in the assembly of the RNAi enzyme complex. Cell.

[36] Wang B., Li S., Qi H.H., Chowdhury D., Shi Y., Novina C.D. (2009). Distinct passenger strand and mRNA cleavage activities of human argonaute proteins. Nat. Struct. Mol. Biol..

[37] Vaucheret H., Vazquez F., Crete P., Bartel D.P. (2004). The action of argonaute1 in the miRNA pathway and its regulation by the miRNA pathway are crucial for plant development. Genes Dev..

[38] Filipowicz W., Bhattacharyya S.N., Sonenberg N. (2008). Mechanisms of post-transcriptional regulation by microRNAs: Are the answers in sight?. Nat. Rev. Genet..

[39] Pillai R.S., Bhattacharyya S.N., Artus C.G., Zoller T., Cougot N., Basyuk E., Bertrand E., Filipowicz W. (2005). Inhibition of translational initiation by let-7 microRNA in human cells. Science.

[40] Humphreys D.T., Westman B.J., Martin D.I., Preiss T. (2005). MicroRNAs control translation initiation by inhibiting eukaryotic initiation factor 4e/cap and poly(a) tail function. Proc. Natl. Acad. Sci. USA.

[41] Richter J.D., Sonenberg N. (2005). Regulation of cap-dependent translation by eif4e inhibitory proteins. Nature.

[42] Chendrimada T.P., Finn K.J., Ji X., Baillat D., Gregory R.I., Liebhaber S.A., Pasquinelli A.E., Shiekhattar R. (2007). MicroRNA silencing through risc recruitment of eif6. Nature.

[43] Maroney P.A., Yu Y., Fisher J., Nilsen T.W. (2006). Evidence that microRNAs are associated with translating messenger RNAs in human cells. Nat. Struct. Mol. Biol..

[44] Parker R., Song H. (2004). The enzymes and control of eukaryotic mRNA turnover. Nat. Struct. Mol. Biol..

[45] Eulalio A., Behm-Ansmant I., Izaurralde E. (2007). P bodies: At the crossroads of post-transcriptional pathways. Nat. Rev. Mol. Cell Biol..

[46] Makarova J.A., Shkurnikov M.U., Wicklein D., Lange T., Samatov T.R., Turchinovich A.A., Tonevitsky A.G. (2016). Intracellular and extracellular microRNA: An update on localization and biological role. Prog. Histochem. Cytochem..

[47] Burza S., Croft S.L., Boelaert M. (2018). Leishmaniasis. Lancet.

[48] Schewach-Millet M., Kahana M., Ronnen M., Yuzuk S. (1986). Mucosal involvement of cutaneous leishmaniasis. Int. J. Dermatol..

[49] WHO. http://www.who.int/en/news-room/fact-sheets/detail/leishmaniasis.

[50] Sacks D., Kamhawi S. (2001). Molecular aspects of parasite-vector and vector-host interactions in leishmaniasis. Annu. Rev. Microbiol..

[51] Teixeira D.E., Benchimol M., Rodrigues J.C., Crepaldi P.H., Pimenta P.F., de Souza W. (2013). The cell biology of leishmania: How to teach using animations. PLoS Pathog..

[52] Gregory D.J., Olivier M. (2005). Subversion of host cell signalling by the protozoan parasite leishmania. Parasitology.

[53] Nathan C., Shiloh M.U. (2000). Reactive oxygen and nitrogen intermediates in the relationship between mammalian hosts and microbial pathogens. Proc. Natl. Acad. Sci. USA.

[54] Mosser D.M., Edwards J.P. (2008). Exploring the full spectrum of macrophage activation. Nat. Rev. Immunol..

[55] Scott P., Novais F.O. (2016). Cutaneous leishmaniasis: Immune responses in protection and pathogenesis. Nat. Rev. Immunol..

[56] Frank B., Marcu A., de Oliveira Almeida Petersen A.L., Weber H., Stigloher C., Mottram J.C., Scholz C.J., Schurigt U. (2015). Autophagic digestion of leishmania major by host macrophages is associated with differential expression of bnip3, ctse, and the miRNAs mir-101c, mir-129, and mir-210. Parasites Vectors.

[57] Mukherjee B., Paul J., Mukherjee S., Mukhopadhyay R., Das S., Naskar K., Sundar S., Dujardin J.C., Saha B., Roy S. (2015). Antimony-resistant leishmania donovani exploits mir-466i to deactivate host myd88 for regulating il-10/il-12 levels during early hours of infection. J. Immunol..

[58] Lemaire J., Mkannez G., Guerfali F.Z., Gustin C., Attia H., Sghaier R.M., Sysco C., Dellagi K., Laouini D., Renard P. (2013). MicroRNA expression profile in human macrophages in response to leishmania major infection. PLoS Negl. Trop. Dis..

[59] Ghosh J., Bose M., Roy S., Bhattacharyya S.N. (2013). Leishmania donovani targets dicer1 to downregulate mir-122, lower serum cholesterol, and facilitate murine liver infection. Cell Host Microbe.

[60] Geraci N.S., Tan J.C., McDowell M.A. (2015). Characterization of microRNA expression profiles in leishmania-infected human phagocytes. Parasite Immunol..

[61] Muxel S.M., Laranjeira-Silva M.F., Zampieri R.A., Floeter-Winter L.M. (2017). Leishmania (leishmania) amazonensis induces macrophage mir-294 and mir-721 expression and modulates infection by targeting nos2 and l-arginine metabolism. Sci. Rep..

[62] Bragato J.P., Melo L.M., Venturin G.L., Rebech G.T., Garcia L.E., Lopes F.L., de Lima V.M.F. (2018). Relationship of peripheral blood mononuclear cells miRNA expression and parasitic load in canine visceral leishmaniasis. PLoS ONE.

[63] Tiwari N., Kumar V., Gedda M.R., Singh A.K., Singh V.K., Gannavaram S., Singh S.P., Singh R.K. (2017). Identification and characterization of miRNAs in response to leishmania donovani infection: Delineation of their roles in macrophage dysfunction. Front. Microbiol..

[64] Corraliza I.M., Soler G., Eichmann K., Modolell M. (1995). Arginase induction by suppressors of nitric oxide synthesis (IL-4, IL-10 and PGE2) in murine bone-marrow-derived macrophages. Biochem. Biophys. Res. Commun..

[65] Munder M., Eichmann K., Modolell M. (1998). Alternative metabolic states in murine macrophages reflected by the nitric oxide synthase/arginase balance: Competitive regulation by cd4+ t cells correlates with th1/th2 phenotype. J. Immunol..

[66] Wanasen N., Soong L. (2008). L-arginine metabolism and its impact on host immunity against leishmania infection. Immunol. Res..

[67] Mantovani A., Sica A., Sozzani S., Allavena P., Vecchi A., Locati M. (2004). The chemokine system in diverse forms of macrophage activation and polarization. Trends Immunol..

[68] Wang N., Liang H., Zen K. (2014). Molecular mechanisms that influence the macrophage m1-m2 polarization balance. Front. Immunol..

[69] Hrabak A., Bajor T., Temesi A., Meszaros G. (1996). The inhibitory effect of nitrite, a stable product of nitric oxide (no) formation, on arginase. FEBS Lett..

[70] Boucher J.L., Moali C., Tenu J.P. (1999). Nitric oxide biosynthesis, nitric oxide synthase inhibitors and arginase competition for l-arginine utilization. Cell Mol. Life Sci..

[71] Verreck F.A., de Boer T., Langenberg D.M., Hoeve M.A., Kramer M., Vaisberg E., Kastelein R., Kolk A., de Waal-Malefyt R., Ottenhoff T.H. (2004). Human il-23-producing type 1 macrophages promote but il-10-producing type 2 macrophages subvert immunity to (myco)bacteria. Proc. Natl. Acad. Sci. USA.

[72] Martinez F.O., Helming L., Gordon S. (2009). Alternative activation of macrophages: An immunologic functional perspective. Annu. Rev. Immunol..

[73] Li H., Jiang T., Li M.Q., Zheng X.L., Zhao G.J. (2018). Transcriptional regulation of macrophages polarization by microRNAs. Front. Immunol..

[74] Lima-Junior D.S., Costa D.L., Carregaro V., Cunha L.D., Silva A.L., Mineo T.W., Gutierrez F.R., Bellio M., Bortoluci K.R., Flavell R.A. (2013). Inflammasome-derived il-1beta production induces nitric oxide-mediated resistance to leishmania. Nat. Med..

[75] Aoki J.I., Muxel S.M., Zampieri R.A., Müller K.E., Nerland A.H., Floeter-Winter L.M. (2019). Differential immune response modulation in early leishmania amazonensis infection of balb/c and c57bl/6 macrophages based on transcriptome profiles. Sci. Rep..

[76] Zea A.H., Rodriguez P.C., Culotta K.S., Hernandez C.P., DeSalvo J., Ochoa J.B., Park H.J., Zabaleta J., Ochoa A.C. (2004). L-arginine modulates cd3zeta expression and t cell function in activated human t lymphocytes. Cell Immunol..

[77] Rodriguez P.C., Zea A.H., DeSalvo J., Culotta K.S., Zabaleta J., Quiceno D.G., Ochoa J.B., Ochoa A.C. (2003). L-arginine consumption by macrophages modulates the expression of cd3 zeta chain in t lymphocytes. J. Immunol..

[78] Kishikawa T., Otsuka M., Tan P.S., Ohno M., Sun X., Yoshikawa T., Shibata C., Takata A., Kojima K., Takehana K. (2015). Decreased mir122 in hepatocellular carcinoma leads to chemoresistance with increased arginine. Oncotarget.

[79] Dunand-Sauthier I., Irla M., Carnesecchi S., Seguin-Estevez Q., Vejnar C.E., Zdobnov E.M., Santiago-Raber M.L., Reith W. (2014). Repression of arginase-2 expression in dendritic cells by microRNA-155 is critical for promoting t cell proliferation. J. Immunol..

[80] Fernandes J.C.R., Aoki J.I., Acuña S.M., Zampieri R.A., Markus R.P., Floeter-Winter L.M., Muxel S.M. (2019). Melatonin and leishmania amazonensis infection altered mir-294, mir-30e, and mir-302d impacting on tnf, mcp-1, and nos2 expression. Front. Cell. Infect. Microbiol..

[81] Guerfali F.Z., Laouini D., Guizani-Tabbane L., Ottones F., Ben-Aissa K., Benkahla A., Manchon L., Piquemal D., Smandi S., Mghirbi O. (2008). Simultaneous gene expression profiling in human macrophages infected with leishmania major parasites using sage. BMC Genom..

[82] Degrossoli A., Bosetto M.C., Lima C.B., Giorgio S. (2007). Expression of hypoxia-inducible factor 1alpha in mononuclear phagocytes infected with leishmania amazonensis. Immunol. Lett..

[83] Kumar V., Kumar A., Das S., Kumar A., Abhishek K., Verma S., Mandal A., Singh R.K., Das P. (2018). Leishmania donovani activates hypoxia inducible factor-1alpha and mir-210 for survival in macrophages by downregulation of nf-kappab mediated pro-inflammatory immune response. Front. Microbiol..

[84] Ismail N., Kaul A., Bhattacharya P., Gannavaram S., Nakhasi H.L. (2017). Immunization with live attenuated leishmania donovani centrin^−/−^ parasites is efficacious in asymptomatic infection. Front. Immunol..

[85] Lago T.S., Silva J.A., Lago E.L., Carvalho E.M., Zanette D.L., Castellucci L.C. (2018). The miRNA 361-3p, a regulator of gzmb and tnf is associated with therapeutic failure and longer time healing of cutaneous leishmaniasis caused by l. (viannia) braziliensis. Front. Immunol..

[86] Crauwels P., Bohn R., Thomas M., Gottwalt S., Jackel F., Kramer S., Bank E., Tenzer S., Walther P., Bastian M. (2015). Apoptotic-like leishmania exploit the host’s autophagy machinery to reduce t-cell-mediated parasite elimination. Autophagy.

[87] Dias B.R.S., de Souza C.S., Almeida N.J., Lima J.G.B., Fukutani K.F., Dos Santos T.B.S., Franca-Cost J., Brodskyn C.I., de Menezes J.P.B., Colombo M.I. (2018). Autophagic induction greatly enhances leishmania major intracellular survival compared to leishmania amazonensis in cba/j-infected macrophages. Front. Microbiol..

[88] Singh A.K., Pandey R.K., Shaha C., Madhubala R. (2016). MicroRNA expression profiling of leishmania donovani-infected host cells uncovers the regulatory role of mir30a-3p in host autophagy. Autophagy.

[89] Tolouei S., Hejazi S.H., Ghaedi K., Khamesipour A., Hasheminia S.J. (2013). Tlr2 and tlr4 in cutaneous leishmaniasis caused by leishmania major. Scand. J. Immunol..

[90] Muxel S.M., Acuna S.M., Aoki J.I., Zampieri R.A., Floeter-Winter L.M. (2018). Toll-like receptor and miRNA-let-7e expression alter the inflammatory response in leishmania amazonensis-infected macrophages. Front. Immunol..

[91] Elizabeth M.C., Hernandez de la Cruz O.N., Mauricio C.A. (2016). Infection of j774a.1 with different mycobacterium species induces differential immune and miRNA-related responses. Microbiol. Immunol..

[92] Kalantari P., Harandi O.F., Agarwal S., Rus F., Kurt-Jones E.A., Fitzgerald K.A., Caffrey D.R., Golenbock D.T. (2017). Mir-718 represses proinflammatory cytokine production through targeting phosphatase and tensin homolog (pten). J. Biol. Chem..

[93] Colineau L., Lambertz U., Fornes O., Wasserman W.W., Reiner N.E. (2018). C-myc is a novel leishmania virulence factor by proxy that targets the host miRNA system and is essential for survival in human macrophages. J. Biol.Chem..

[94] Abrudan J., Ramalho-Ortigao M., O’Neil S., Stayback G., Wadsworth M., Bernard M., Shoue D., Emrich S., Lawyer P., Kamhawi S. (2013). The characterization of the phlebotomus papatasi transcriptome. Insect Mol. Biol..

[95] Ferreira F.V., Aguiar E., Olmo R.P., de Oliveira K.P.V., Silva E.G., Sant’Anna M.R.V., Gontijo N.F., Kroon E.G., Imler J.L., Marques J.T. (2018). The small non-coding RNA response to virus infection in the leishmania vector lutzomyia longipalpis. PLoS Negl. Trop. Dis..

[96] Rossi E., Bongiorno G., Ciolli E., Di Muccio T., Scalone A., Gramiccia M., Gradoni L., Maroli M. (2008). Seasonal phenology, host-blood feeding preferences and natural leishmania infection of phlebotomus perniciosus (diptera, psychodidae) in a high-endemic focus of canine leishmaniasis in Rome province, Italy. Acta Trop..

[97] Yang Z., Wu Y. (2019). Improved annotation of lutzomyia longipalpis genome using bioinformatics analysis. PeerJ.

[98] Petrella V., Aceto S., Musacchia F., Colonna V., Robinson M., Benes V., Cicotti G., Bongiorno G., Gradoni L., Volf P. (2015). De novo assembly and sex-specific transcriptome profiling in the sand fly phlebotomus perniciosus (diptera, phlebotominae), a major old world vector of leishmania infantum. BMC Genom..

[99] Robinson K.A., Beverley S.M. (2003). Improvements in transfection efficiency and tests of RNA interference (RNAi) approaches in the protozoan parasite leishmania. Mol. Biochem. Parasitol..

[100] Rastrojo A., Carrasco-Ramiro F., Martin D., Crespillo A., Reguera R.M., Aguado B., Requena J.M. (2013). The transcriptome of leishmania major in the axenic promastigote stage: Transcript annotation and relative expression levels by RNA-seq. BMC Genom..

[101] Fernandes M.C., Dillon L.A., Belew A.T., Bravo H.C., Mosser D.M., El-Sayed N.M. (2016). Dual transcriptome profiling of leishmania-infected human macrophages reveals distinct reprogramming signatures. MBio.

[102] Aoki J.I., Muxel S.M., Zampieri R.A., Laranjeira-Silva M.F., Muller K.E., Nerland A.H., Floeter-Winter L.M. (2017). RNA-seq transcriptional profiling of leishmania amazonensis reveals an arginase-dependent gene expression regulation. PLoS Negl. Trop. Dis..

[103] World Health Organization. http://www.who.int/wer/2015/wer9006.pdf?ua=1.

[104] Bonney K.M. (2014). Chagas disease in the 21st century: A public health success or an emerging threat?. Parasite.

[105] Lidani K.C.F., Andrade F.A., Bavia L., Damasceno F.S., Beltrame M.H., Messias-Reason I.J., Sandri T.L. (2019). Chagas disease: From discovery to a worldwide health problem. Front. Public. Health.

[106] Lee B.Y., Bacon K.M., Bottazzi M.E., Hotez P.J. (2013). Global economic burden of chagas disease: A computational simulation model. Lancet Infect. Dis..

[107] Nagajyothi F., Machado F.S., Burleigh B.A., Jelicks L.A., Scherer P.E., Mukherjee S., Lisanti M.P., Weiss L.M., Garg N.J., Tanowitz H.B. (2012). Mechanisms of trypanosoma cruzi persistence in chagas disease. Cell. Microbiol..

[108] Rassi A., Rassi A., Marin-Neto J.A. (2010). Chagas disease. Lancet.

[109] Basso B. (2013). Modulation of immune response in experimental chagas disease. World J. Exp. Med..

[110] Zingales B., Andrade S.G., Briones M.R., Campbell D.A., Chiari E., Fernandes O., Guhl F., Lages-Silva E., Macedo A.M., Machado C.R. (2009). A new consensus for trypanosoma cruzi intraspecific nomenclature: Second revision meeting recommends tci to tcvi. Mem. Inst. Oswaldo Cruz.

[111] Kratz J.M., Garcia Bournissen F., Forsyth C.J., Sosa-Estani S. (2018). Clinical and pharmacological profile of benznidazole for treatment of chagas disease. Expert Rev. Clin. Pharmacol..

[112] Ferreira L.R., Frade A.F., Baron M.A., Navarro I.C., Kalil J., Chevillard C., Cunha-Neto E. (2014). Interferon-gamma and other inflammatory mediators in cardiomyocyte signaling during chagas disease cardiomyopathy. World J. Cardiol..

[113] Andrade D.V., Gollob K.J., Dutra W.O. (2014). Acute chagas disease: New global challenges for an old neglected disease. PLoS Negl. Trop. Dis..

[114] Abel L.C., Rizzo L.V., Ianni B., Albuquerque F., Bacal F., Carrara D., Bocchi E.A., Teixeira H.C., Mady C., Kalil J. (2001). Chronic chagas’ disease cardiomyopathy patients display an increased ifn-gamma response to trypanosoma cruzi infection. J. Autoimmun..

[115] Nogueira L.G., Santos R.H., Fiorelli A.I., Mairena E.C., Benvenuti L.A., Bocchi E.A., Stolf N.A., Kalil J., Cunha-Neto E. (2014). Myocardial gene expression of T-bet, GATA-3, Ror-γt, FoxP3, and hallmark cytokines in chronic chagas disease cardiomyopathy: An essentially unopposed TH1-type response. Mediat. Inflamm..

[116] Navarro I.C., Ferreira F.M., Nakaya H.I., Baron M.A., Vilar-Pereira G., Pereira I.R., Silva A.M., Real J.M., De Brito T., Chevillard C. (2015). MicroRNA transcriptome profiling in heart of trypanosoma cruzi-infected mice: Parasitological and cardiological outcomes. PLoS Negl. Trop. Dis..

[117] Ferreira L.R.P., Ferreira F.M., Laugier L., Cabantous S., Navarro I.C., da Silva Candido D., Rigaud V.C., Real J.M., Pereira G.V., Pereira I.R. (2017). Integration of miRNA and gene expression profiles suggest a role for miRNAs in the pathobiological processes of acute trypanosoma cruzi infection. Sci. Rep..

[118] Cunha-Neto E., Dzau V.J., Allen P.D., Stamatiou D., Benvenutti L., Higuchi M.L., Koyama N.S., Silva J.S., Kalil J., Liew C.C. (2005). Cardiac gene expression profiling provides evidence for cytokinopathy as a molecular mechanism in chagas’ disease cardiomyopathy. Am. J. Pathol..

[119] Meng L.D., Meng A.C., Zhu Q., Jia R.Y., Kong Q.Z. (2015). Effect of microRNA-208a on mitochondrial apoptosis of cardiomyocytes of neonatal rats. Asian Pac. J. Trop. Med..

[120] Shyu K.G., Wang B.W., Cheng W.P., Lo H.M. (2015). MicroRNA-208a increases myocardial endoglin expression and myocardial fibrosis in acute myocardial infarction. Can. J. Cardiol..

[121] Nonaka C.K.V., Macedo C.T., Cavalcante B.R.R., Alcantara A.C., Silva D.N., Bezerra M.D.R., Caria A.C.I., Tavora F.R.F., Neto J.D.S., Noya-Rabelo M.M. (2019). Circulating miRNAs as potential biomarkers associated with cardiac remodeling and fibrosis in chagas disease cardiomyopathy. Int. J. Mol. Sci..

[122] Zhou X., Li Q., Xu J., Zhang X., Zhang H., Xiang Y., Fang C., Wang T., Xia S., Zhang Q. (2016). The aberrantly expressed mir-193b-3p contributes to preeclampsia through regulating transforming growth factor-beta signaling. Sci. Rep..

[123] Gu N., You L., Shi C., Yang L., Pang L., Cui X., Ji C., Zheng W., Guo X. (2016). Expression of mir-199a-3p in human adipocytes is regulated by free fatty acids and adipokines. Mol. Med. Rep..

[124] Savino W. (2010). Intrathymic t cell migration is a multivectorial process under a complex neuroendocrine control. Neuroimmunomodulation.

[125] Linhares-Lacerda L., Palu C.C., Ribeiro-Alves M., Paredes B.D., Morrot A., Garcia-Silva M.R., Cayota A., Savino W. (2015). Differential expression of microRNAs in thymic epithelial cells from trypanosoma cruzi acutely infected mice: Putative role in thymic atrophy. Front. Immunol..

[126] Bayer-Santos E., Lima F.M., Ruiz J.C., Almeida I.C., da Silveira J.F. (2014). Characterization of the small RNA content of trypanosoma cruzi extracellular vesicles. Mol. Biochem. Parasitol..

[127] Garcia-Silva M.R., Cabrera-Cabrera F., das Neves R.F., Souto-Padron T., de Souza W., Cayota A. (2014). Gene expression changes induced by trypanosoma cruzi shed microvesicles in mammalian host cells: Relevance of tRNA-derived halves. Biomed. Res. Int..

[128] Franzen O., Arner E., Ferella M., Nilsson D., Respuela P., Carninci P., Hayashizaki Y., Aslund L., Andersson B., Daub C.O. (2011). The short non-coding transcriptome of the protozoan parasite trypanosoma cruzi. PLoS Negl. Trop. Dis..

[129] Fernandez-Calero T., Garcia-Silva R., Pena A., Robello C., Persson H., Rovira C., Naya H., Cayota A. (2015). Profiling of small RNA cargo of extracellular vesicles shed by trypanosoma cruzi reveals a specific extracellular signature. Mol. Biochem. Parasitol..

[130] World Health Organization. http://www.who.int/ith/diseases/trypanosomiasis/en/.

[131] Ralston K.S., Kabututu Z.P., Melehani J.H., Oberholzer M., Hill K.L. (2009). The trypanosoma brucei flagellum: Moving parasites in new directions. Annu. Rev. Microbiol..

[132] Lythgoe K.A., Morrison L.J., Read A.F., Barry J.D. (2007). Parasite-intrinsic factors can explain ordered progression of trypanosome antigenic variation. Proc. Natl. Acad. Sci. USA.

[133] Rico E., Rojas F., Mony B.M., Szoor B., Macgregor P., Matthews K.R. (2013). Bloodstream form pre-adaptation to the tsetse fly in trypanosoma brucei. Front. Cell. Infect. Microbiol..

[134] Lueong S., Leong S., Simo G., Camara M., Jamonneau V., Kabore J., Ilboudo H., Bucheton B., Hoheisel J.D., Clayton C. (2013). The miRNA and mRNA signatures of peripheral blood cells in humans infected with trypanosoma brucei gambiense. PLoS ONE.

[135] Kolev N.G., Franklin J.B., Carmi S., Shi H., Michaeli S., Tschudi C. (2010). The transcriptome of the human pathogen trypanosoma brucei at single-nucleotide resolution. PLoS Pathog..

[136] Liniger M., Bodenmuller K., Pays E., Gallati S., Roditi I. (2001). Overlapping sense and antisense transcription units in trypanosoma brucei. Mol. Microbiol..

[137] Mallick B., Ghosh Z., Chakrabarti J. (2008). MicroRNA switches in trypanosoma brucei. Biochem. Biophys. Res. Commun..

[138] Boothroyd J.C., Grigg M.E. (2002). Population biology of toxoplasma gondii and its relevance to human infection: Do different strains cause different disease?. Curr. Opin. Microbiol..

[139] Montoya J.G., Liesenfeld O. (2004). Toxoplasmosis. Lancet.

[140] Parlog A., Schluter D., Dunay I.R. (2015). Toxoplasma gondii-induced neuronal alterations. Parasite Immunol..

[141] Ehmen H.G., Luder C.G.K. (2019). Long-term impact of toxoplasma gondii infection on human monocytes. Front. Cell. Infect. Microbiol..

[142] Hargrave K.E., Woods S., Millington O., Chalmers S., Westrop G.D., Roberts C.W. (2019). Multi-omics studies demonstrate toxoplasma gondii-induced metabolic reprogramming of murine dendritic cells. Front. Cell. Infect. Microbiol..

[143] Saadatnia G., Golkar M. (2012). A review on human toxoplasmosis. Scand. J. Infect. Dis..

[144] Da Silva R.C., Langoni H. (2009). Toxoplasma gondii: Host-parasite interaction and behavior manipulation. Parasitol. Res..

[145] Zeiner G.M., Norman K.L., Thomson J.M., Hammond S.M., Boothroyd J.C. (2010). Toxoplasma gondii infection specifically increases the levels of key host microRNAs. PLoS ONE.

[146] O’Connor L., Strasser A., O’Reilly L.A., Hausmann G., Adams J.M., Cory S., Huang D.C. (1998). Bim: A novel member of the bcl-2 family that promotes apoptosis. EMBO J..

[147] Goebel S., Gross U., Luder C.G. (2001). Inhibition of host cell apoptosis by toxoplasma gondii is accompanied by reduced activation of the caspase cascade and alterations of poly(adp-ribose) polymerase expression. J. Cell Sci..

[148] Ota A., Tagawa H., Karnan S., Tsuzuki S., Karpas A., Kira S., Yoshida Y., Seto M. (2004). Identification and characterization of a novel gene, c13orf25, as a target for 13q31-q32 amplification in malignant lymphoma. Cancer Res..

[149] Kuo G., Wu C.Y., Yang H.Y. (2019). Mir-17-92 cluster and immunity. J. Formos. Med. Assoc..

[150] O’Donnell K.A., Wentzel E.A., Zeller K.I., Dang C.V., Mendell J.T. (2005). C-myc-regulated microRNAs modulate e2f1 expression. Nature.

[151] Baumjohann D. (2018). Diverse functions of mir-17-92 cluster microRNAs in t helper cells. Cancer Lett..

[152] Xiao C., Srinivasan L., Calado D.P., Patterson H.C., Zhang B., Wang J., Henderson J.M., Kutok J.L., Rajewsky K. (2008). Lymphoproliferative disease and autoimmunity in mice with increased mir-17-92 expression in lymphocytes. Nat. Immunol..

[153] Koralov S.B., Muljo S.A., Galler G.R., Krek A., Chakraborty T., Kanellopoulou C., Jensen K., Cobb B.S., Merkenschlager M., Rajewsky N. (2008). Dicer ablation affects antibody diversity and cell survival in the b lymphocyte lineage. Cell.

[154] Hou Z., Liu D., Su S., Wang L., Zhao Z., Ma Y., Li Q., Jia C., Xu J., Zhou Y. (2019). Comparison of splenocyte microRNA expression profiles of pigs during acute and chronic toxoplasmosis. BMC Genom..

[155] Cannella D., Brenier-Pinchart M.P., Braun L., van Rooyen J.M., Bougdour A., Bastien O., Behnke M.S., Curt R.L., Curt A., Saeij J.P. (2014). Mir-146a and mir-155 delineate a microRNA fingerprint associated with toxoplasma persistence in the host brain. Cell Rep..

[156] Jia B., Chang Z., Wei X., Lu H., Yin J., Jiang N., Chen Q. (2014). Plasma microRNAs are promising novel biomarkers for the early detection of toxoplasma gondii infection. Parasites Vectors.

[157] Hu R.S., He J.J., Elsheikha H.M., Zhang F.K., Zou Y., Zhao G.H., Cong W., Zhu X.Q. (2018). Differential brain microRNA expression profiles after acute and chronic infection of mice with toxoplasma gondii oocysts. Front. Microbiol..

[158] He J.J., Ma J., Wang J.L., Xu M.J., Zhu X.Q. (2016). Analysis of miRNA expression profiling in mouse spleen affected by acute toxoplasma gondii infection. Infect. Genet. Evol..

[159] Xiao J., Li Y., Prandovszky E., Karuppagounder S.S., Talbot C.C., Dawson V.L., Dawson T.M., Yolken R.H. (2014). MicroRNA-132 dysregulation in toxoplasma gondii infection has implications for dopamine signaling pathway. Neuroscience.

[160] Ngo H.M., Zhou Y., Lorenzi H., Wang K., Kim T.K., Zhou Y., El Bissati K., Mui E., Fraczek L., Rajagopala S.V. (2017). Toxoplasma modulates signature pathways of human epilepsy, neurodegeneration & cancer. Sci. Rep..

[161] Cong W., Zhang X.X., He J.J., Li F.C., Elsheikha H.M., Zhu X.Q. (2017). Global miRNA expression profiling of domestic cat livers following acute toxoplasma gondii infection. Oncotarget.

[162] World Health Organization. http://www.who.int/news-room/fact-sheets/detail/malaria.

[163] Engwerda C.R., Beattie L., Amante F.H. (2005). The importance of the spleen in malaria. Trends Parasitol..

[164] Wunderlich F., Al-Quraishy S., Dkhil M.A. (2014). Liver-inherent immune system: Its role in blood-stage malaria. Front. Microbiol..

[165] Hentzschel F., Hammerschmidt-Kamper C., Borner K., Heiss K., Knapp B., Sattler J.M., Kaderali L., Castoldi M., Bindman J.G., Malato Y. (2014). Aav8-mediated in vivo overexpression of mir-155 enhances the protective capacity of genetically attenuated malarial parasites. Mol. Ther..

[166] Dkhil M.A., Al-Quraishy S.A., Abdel-Baki A.S., Delic D., Wunderlich F. (2016). Differential miRNA expression in the liver of balb/c mice protected by vaccination during crisis of plasmodium chabaudi blood-stage malaria. Front. Microbiol..

[167] Martin-Alonso A., Cohen A., Quispe-Ricalde M.A., Foronda P., Benito A., Berzosa P., Valladares B., Grau G.E. (2018). Differentially expressed microRNAs in experimental cerebral malaria and their involvement in endocytosis, adherens junctions, foxo and tgf-beta signalling pathways. Sci. Rep..

[168] El-Assaad F., Hempel C., Combes V., Mitchell A.J., Ball H.J., Kurtzhals J.A., Hunt N.H., Mathys J.M., Grau G.E. (2011). Differential microRNA expression in experimental cerebral and noncerebral malaria. Infect. Immun..

[169] Chamnanchanunt S., Kuroki C., Desakorn V., Enomoto M., Thanachartwet V., Sahassananda D., Sattabongkot J., Jenwithisuk R., Fucharoen S., Svasti S. (2015). Downregulation of plasma mir-451 and mir-16 in plasmodium vivax infection. Exp. Parasitol..

[170] Glinsky G.V. (2008). Snp-guided microRNA maps (mirmaps) of 16 common human disorders identify a clinically accessible therapy reversing transcriptional aberrations of nuclear import and inflammasome pathways. Cell Cycle.

[171] van Loon W., Gai P.P., Hamann L., Bedu-Addo G., Mockenhaupt F.P. (2019). MiRNA-146a polymorphism increases the odds of malaria in pregnancy. Malar. J..

[172] Taganov K.D., Boldin M.P., Chang K.J., Baltimore D. (2006). Nf-kappab-dependent induction of microRNA mir-146, an inhibitor targeted to signaling proteins of innate immune responses. Proc. Natl. Acad. Sci. USA.

[173] Longley R., Smith C., Fortin A., Berghout J., McMorran B., Burgio G., Foote S., Gros P. (2011). Host resistance to malaria: Using mouse models to explore the host response. Mamm. Genome.

[174] Freitas do Rosario A.P., Lamb T., Spence P., Stephens R., Lang A., Roers A., Muller W., O’Garra A., Langhorne J. (2012). Il-27 promotes il-10 production by effector th1 cd4+ t cells: A critical mechanism for protection from severe immunopathology during malaria infection. J. Immunol..

[175] Stephens R., Culleton R.L., Lamb T.J. (2012). The contribution of plasmodium chabaudi to our understanding of malaria. Trends Parasitol..

[176] Muxel S.M., Freitas do Rosario A.P., Zago C.A., Castillo-Mendez S.I., Sardinha L.R., Rodriguez-Malaga S.M., Camara N.O., Alvarez J.M., Lima M.R. (2011). The spleen cd4+ t cell response to blood-stage plasmodium chabaudi malaria develops in two phases characterized by different properties. PLoS ONE.

[177] Delic D., Dkhil M., Al-Quraishy S., Wunderlich F. (2011). Hepatic miRNA expression reprogrammed by plasmodium chabaudi malaria. Parasitol. Res..

[178] Mitchell G., Isberg R.R. (2017). Innate immunity to intracellular pathogens: Balancing microbial elimination and inflammation. Cell Host Microbe.

[179] Agop-Nersesian C., Niklaus L., Wacker R., Theo Heussler V. (2018). Host cell cytosolic immune response during plasmodium liver stage development. FEMS Microbiol. Rev..

[180] Vembar S.S., Scherf A., Siegel T.N. (2014). Noncoding RNAs as emerging regulators of plasmodium falciparum virulence gene expression. Curr. Opin. Microbiol..

[181] Guizetti J., Barcons-Simon A., Scherf A. (2016). Trans-acting gc-rich non-coding RNA at var expression site modulates gene counting in malaria parasite. Nucleic Acids Res..

[182] Biryukova I., Ye T., Levashina E. (2014). Transcriptome-wide analysis of microRNA expression in the malaria mosquito anopheles gambiae. BMC Genom..

[183] Winter F., Edaye S., Huttenhofer A., Brunel C. (2007). Anopheles gambiae miRNAs as actors of defence reaction against plasmodium invasion. Nucleic Acids Res..

[184] Jain S., Rana V., Shrinet J., Sharma A., Tridibes A., Sunil S., Bhatnagar R.K. (2014). Blood feeding and plasmodium infection alters the mirnome of anopheles stephensi. PLoS ONE.

[185] Chakraborty C., Sharma A.R., Sharma G., Doss C.G.P., Lee S.S. (2017). Therapeutic miRNA and siRNA: Moving from bench to clinic as next generation medicine. Mol. Ther. Nucleic Acids.

[186] Hewson C., Capraro D., Burdach J., Whitaker N., Morris K.V. (2016). Extracellular vesicle associated long non-coding RNAs functionally enhance cell viability. Non-Coding RNA Res..

